# Hepatocellular Carcinoma With Small Bowel Metastasis and Intussusception: A Case Report and Literature Review

**DOI:** 10.1002/cnr2.70364

**Published:** 2025-11-29

**Authors:** Meng‐Kai Hsu, Po‐Da Chen, Yao‐Ming Wu

**Affiliations:** ^1^ Department of Surgery National Taiwan University Hospital Taipei Taiwan; ^2^ Department of Surgical Oncology National Taiwan University Cancer Center Hospital Taipei Taiwan

**Keywords:** bowel obstruction, gastrointestinal tract metastasis, hepatocellular carcinoma, intussusception, small bowel metastasis, surgical resection

## Abstract

**Background:**

Hepatocellular carcinoma (HCC) is a common primary liver malignancy. Despite advancements in treatment, HCC often has a poor prognosis because of frequent recurrence and metastasis. Hematogenous metastasis to the gastrointestinal tract is uncommon, and metastasis to the small intestine is particularly rare.

**Case:**

We report a case of a 72‐year‐old man with a history of hepatitis C virus and alcohol‐related HCC. In 2017, he was initially managed with atypical hepatectomy of segments 5 and 6. He later developed bone and lung metastases, controlled by sequential systemic therapies. In early 2024, after undergoing a craniotomy for hemorrhagic occipital brain metastasis, he presented with diffuse abdominal pain, fullness, and vomiting. Imaging revealed distal ileal intussusception. A segmental small bowel resection was performed, and a fungating ileal tumor was identified as the lead point. Histology confirmed metastatic HCC (i.e., Hepar‐1 positive). The postoperative course was uneventful, but within 3 months, new metastatic lesions appeared in the stomach and left breast; the latter was treated with a partial mastectomy.

**Conclusion:**

Small intestine metastasis from HCC, although rare, should be considered in patients with gastrointestinal symptoms or unexplained elevated tumor markers. Surgical resection is the most effective treatment approach, with minimally invasive and endoscopic techniques available for select cases.

## 1 Introduction

Hepatocellular carcinoma (HCC) is the most common primary liver malignancy and globally ranks as the fifth and seventh most common cancer in men and women, respectively [1]. In Taiwan, it is the fourth most common cancer and the second leading cause of cancer‐related mortality [2]. Despite advances in surgical techniques and the development of immunotherapy and targeted therapies, the prognosis of HCC remains poor, owing to the high rates of recurrence and metastasis [3]. Extrahepatic metastasis occurs in 13.5%–36.7% of patients, with the lungs, bone, lymph nodes, and adrenal glands being the most frequent sites. Metastases to the peritoneum, brain, and skin, although less common, have also been reported [4]. Hematogenous metastasis of HCC to the gastrointestinal tract is relatively rare, and metastasis to the small intestine is even rarer. In this article, we describe a case of HCC with small intestinal metastasis, presenting with intussusception and bowel obstruction.

## 2 Case Report

In April 2024, a 72‐year‐old man presented to National Taiwan University Hospital (Taipei, Taiwan) emergency department, with a 2‐day history of diffuse abdominal pain and fullness. In November 2017, he was diagnosed with a 7‐cm HCC in segments S5 and S6 with rupture via computed tomography (CT), which also showed liver cirrhosis, no lymphadenopathy, and a patent portal vein. The HCC may have been attributable to hepatitis C virus infection and alcoholism. The patient underwent successful atypical hepatectomy at segments S5 and S6. Every 2–3 months, he underwent regular follow‐up and had normal alpha‐fetoprotein (AFP) levels of 3.4–5.3 ng/mL (normal: < 20 ng/mL).

Annual CT imaging and abdominal sonography revealed no recurrence until February 2020, when progressive lower back pain developed. CT revealed bone and bilateral lung metastases. Bone metastasis at L5 with nerve root compression was managed with surgical decompression, partial tumor excision, fixation, and palliative radiotherapy (RT) (3500 cGy/10 fractions). In March 2020, sorafenib (400 mg/d) was started because of the metastatic disease status.

By July 2020, progressive lung metastasis and Grade 2–3 hand–foot syndrome were noted, prompting a switch to lenvatinib (10 mg/d for 2 weeks, then held for 1 week). Follow‐up CT showed progressive disease with enlargement of the mediastinal lymphadenopathy. In October 2020, regorafenib (80 mg/40 mg QOD) was initiated. The patient tolerated the treatment well with only Grade 1 hand–foot syndrome.

The disease remained stable until November 2021, when MRI showed progression of the L5 bone metastasis. Stereotactic body radiotherapy (SBRT, 1000 cGy/1 fraction) was performed in December 2021. Disease progression led to a switch to atezolizumab (7.5 mg/kg) plus bevacizumab (1200‐mg fixed dose) in August 2022. However, an itchy maculopapular rash developed after 2 weeks. Immune‐related toxicoderma was suspected, and therapy was changed to pembrolizumab (100 mg fixed dose Q3W) in September 2022. After eight cycles, treatment was paused in March 2023 because of abnormal liver function: total bilirubin, 3.05 mg/dL (normal: 0.3–1 mg/dL), aspartate aminotransferase, 235 U/L (normal: 8–31 U/L), and alanine transaminase, 179 U/L (normal: 0–41 U/L).

During systemic therapy, the AFP level remained < 10 ng/mL, despite radiologic progression of disease. The protein induced by vitamin K absence or antagonist‐II (PIVKA‐II) was checked since immunotherapy was carried out; the level improved from approximately 3000 to 1633 mAU/mL (normal: 10–31 mAU/mL) at the end of treatment in March 2023. Within 3 months after treatment cessation, the AFP and PIVKA‐II levels increased to 15.74 ng/mL and 4735.5 mAU/mL, respectively, although imaging in early 2024 showed stable disease. After recurrence, the tumor markers were monitored every 1–2 months, with CT/MRI performed at least every 3 months.

One month before admission, the patient experienced a fall because of dizziness. Brain CT and MRI revealed a new right occipital tumor with hemorrhage, which was managed with craniotomy. Despite an uneventful recovery after the craniotomy, the patient developed left hemiparesis.

**FIGURE 1:**
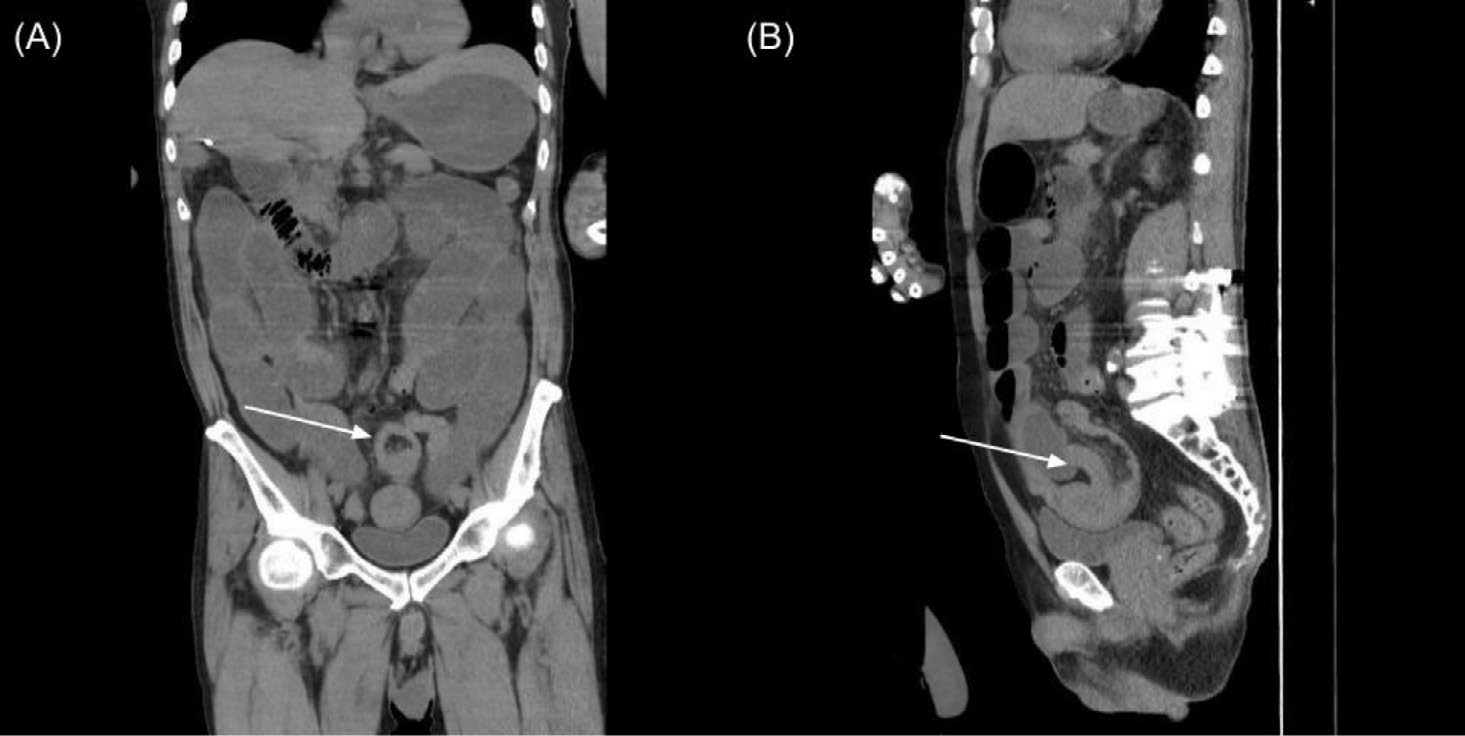
The preoperative computed tomography images show diffuse small bowel ileus with distal ileum intussusception (white arrow) in (A) the coronal view and (B) the sagittal view.

Three days after being discharged from the neurosurgery department, the patient presented with diffuse abdominal fullness, pain, and vomiting. Laboratory test results revealed anemia (hemoglobin, 8.0 g/dL; normal: 13.1–17.2 g/dL), leukocytosis (WBC, 16.76 K/L; normal: 3.25–9.16 K/L), and elevated C‐reactive protein (17.81, mg/dL; normal: < 1 mg/dL) and serum creatinine levels (1.53 mg/dL; normal: 0.6–1.3 mg/dL). CT revealed diffuse small bowel dilation with a transition point in the distal ileum, which suggested intussusception (Figure 1). Initial conservative management with a nasogastric (NG) tube and prokinetics failed, with persistent 500 mL/day bilious NG drainage. Exploratory laparotomy with segmental ileal resection and side‐to‐side anastomosis was performed.

**FIGURE 2:**
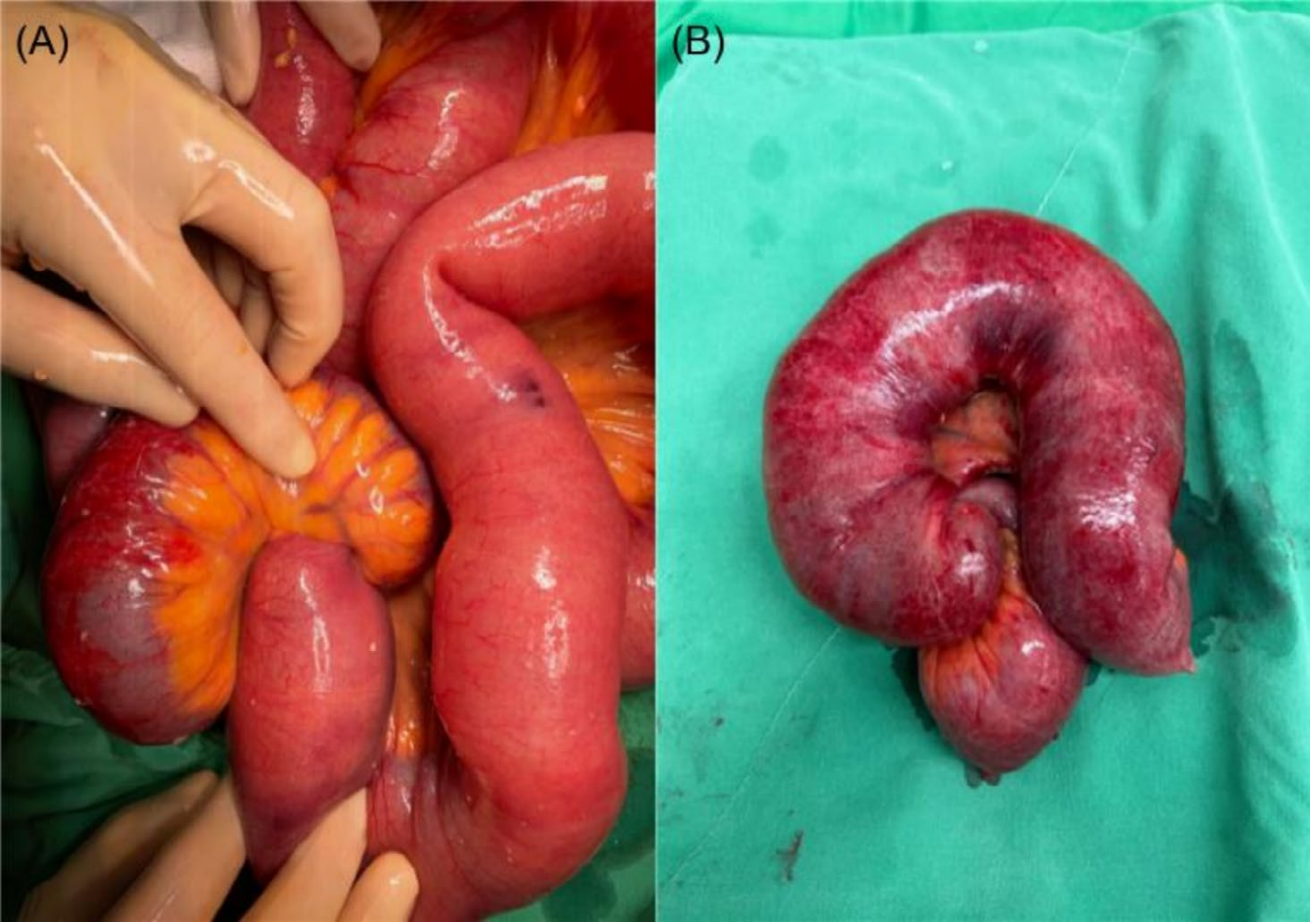
(A) Intraoperative finding of distal ileum intussusception. (B) Small bowel segmental resection.

**FIGURE 3:**
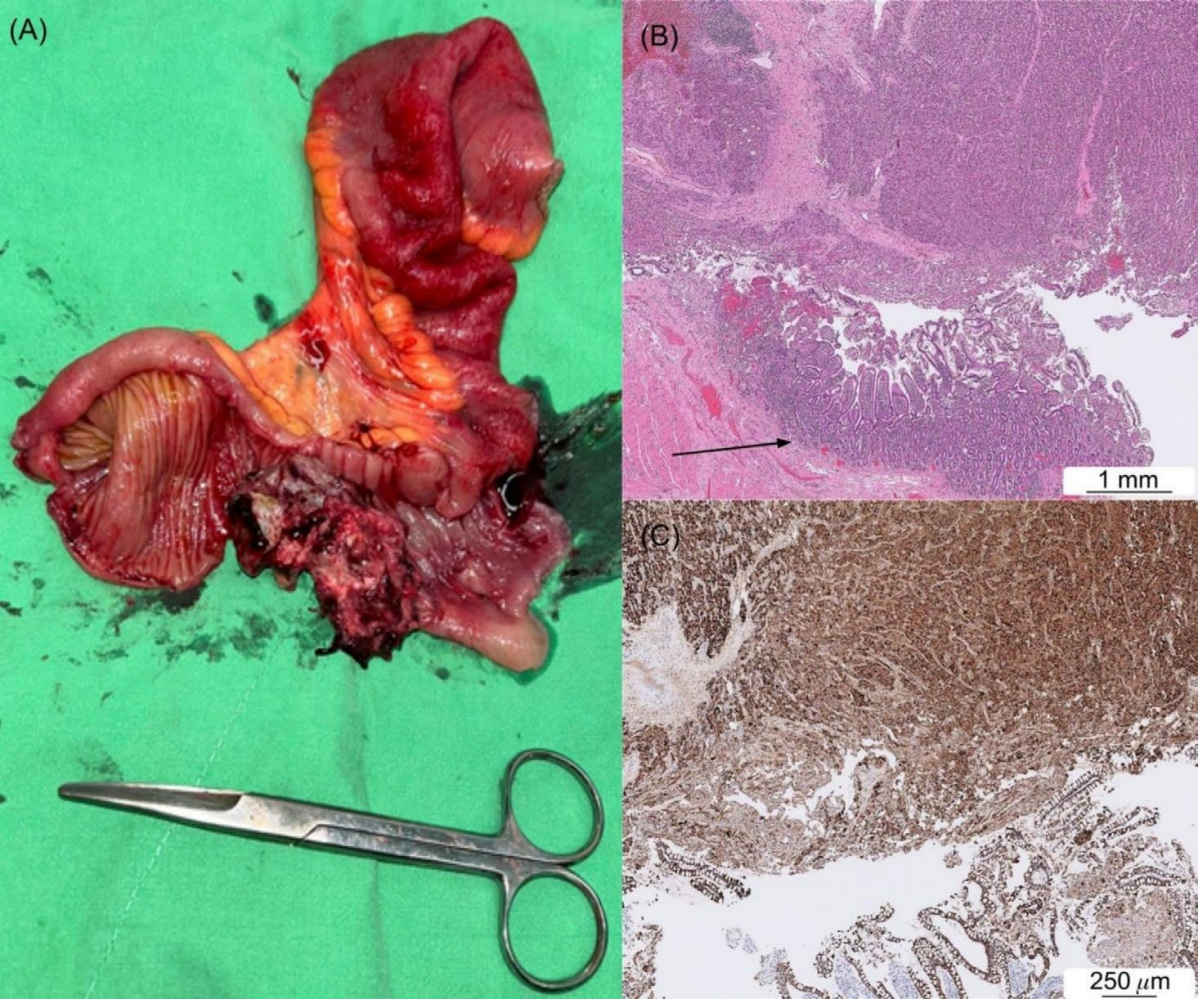
(A) A fungating tumor protruding from the ileum mucosa is the leading point with an erythematous change in the proximal ileum. (B) Histology findings reveal hepatocellular carcinoma metastasis to the small intestine with normal small bowel mucosa (lower part of the figure, black arrow) (HE; magnification, 2.46×) (C) Positive Hepar‐1 staining confirms the diagnosis of HCC metastasis to the small bowel (magnification, 6.26×). HE, hematoxylin and eosin; HCC, hepatocellular carcinoma.

The transition point was identified as an ileoileal intussusception with a fungating tumor protruding from the ileal mucosa as the leading point (Figure 2). Histological analysis confirmed HCC metastasis, based on positive Hepar‐1 staining, and no lymph node metastasis (Figure 3). The postoperative course was relatively uneventful. The patient had mild wound pain and intermittent bloody stool in the first postoperative week, which were controlled with tramadol and an intravenous proton pump inhibitor, respectively. He was discharged on postoperative Day 13.

During the subsequent 3 months, two new HCC metastases were identified. One lesion in the left breast was first documented by a dermatologist in September 2022 as a left chest nodule. The lesion had progressed in size by April 2024. A left partial mastectomy was performed in June 2024. Pathology confirmed metastatic HCC.

**FIGURE 4:**
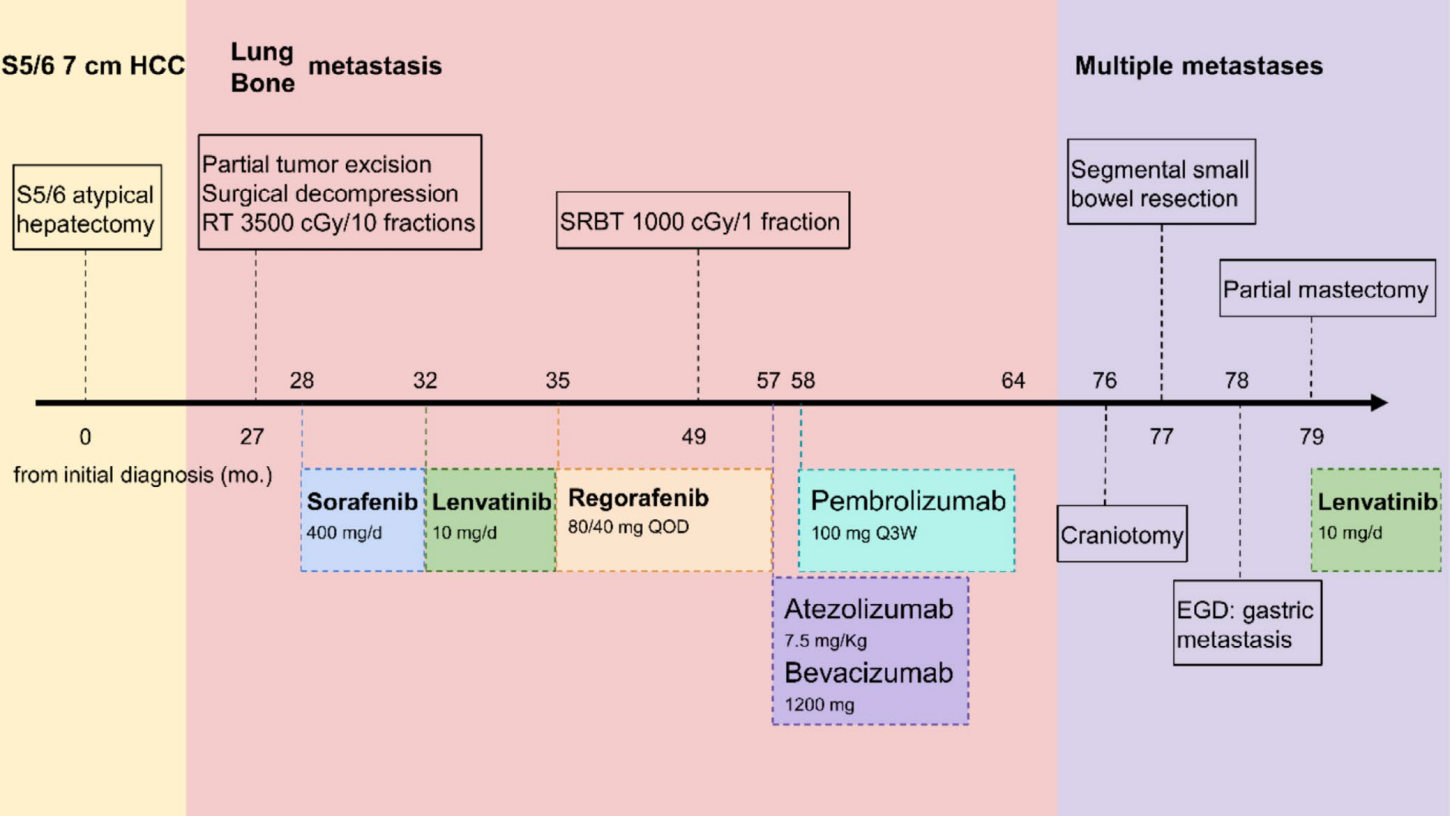
Timeline of the present case. HCC, hepatocellular carcinoma; SBRT, stereotactic body radiotherapy; EGD, esophagogastroduodenoscopy.

In May 2024, another metastasis in the stomach was diagnosed via a follow‐up esophagogastroduodenoscopy (EGD). It presented as an ulcerative, exophytic mass. Target therapy with oral lenvatinib (10 mg/d) has been administered since June 2024. Eighty‐six months after the initial diagnosis, the patient continues to undergo follow‐up. The timeline is presented in Figure 4.

## 3 Discussion

We reported a case of a 72‐year‐old man with HCC who experienced a prolonged disease course culminating in the rare event of small bowel metastasis. He was initially diagnosed with a 7‐cm ruptured HCC and underwent an atypical hepatectomy. Twenty‐seven months after the diagnosis, bone and lung metastases developed and were managed with sequential targeted therapies (i.e., sorafenib, lenvatinib, and regorafenib), followed by immune checkpoint inhibitors (i.e., atezolizumab–bevacizumab and pembrolizumab). Although systemic therapy achieved temporary radiologic stability with an improvement in tumor marker levels, the disease progressed. Fifty months after the first extrahepatic metastasis, he presented with ileoileal intussusception caused by ileal metastasis.

Small bowel metastases from HCC are extremely uncommon, with an incidence of only 0.5%–2%, and are usually associated with rapid deterioration [5, 6]. This case is noteworthy for the exceptionally rare presentation of small bowel metastasis causing intussusception and for the prolonged survival and extended course of extrahepatic disease progression achieved via multiple lines of targeted therapy and immunotherapy.

Hematogenous metastases of HCC to the small intestine are rare. To date, only 15 cases have been reported in the literature (Table 1). In two cases (Cases 12 and 15), metastases were identified only at autopsy. Therefore, the remaining 13 cases were included in the epidemiological analysis, along with the current case (Table 2).

**TABLE 1:** Reported cases of HCC with hematogenous small bowel metastasis.

Case no.	Age (years)	Sex	Symptom	Diagnostic modality	Metastatic site	Systemic treatment	First metastasis interval^a^/small bowel metastasis interval^b^ (months)	Survival (months)^c^	Other metastatic site	Treatment	Authors
1	75	M	IDA	CT	Distal ileum	Sorafenib, regorafenib, nivolumab	0/30	N/A	Lung	Segmental resection	Hong et al. [7]
2	71	M	Intussusception	CT	Ileum, 130 cm distal to the Treitz ligament	Sorafenib	6/70	6	Peritoneum	Segmental resection	Mashiko et al. [8]
3	43	F	Elevated AFP level	^18^F‐FDG PET/CT	Ileum	No treatment	N/A	N/A	Lung	Segmental resection	Yoo et al. [9]
4	76	F	Elevated levels of AFP and PIVKA II	CT hepatic arteriography	Duodenum 2nd portion	No treatment	15/0	N/A	Nil	Duodenal partial resection	Arima et al. [5]
5	60	M	Melena and anemia	Capsule endoscopy	Ileum	Sorafenib	N/A	2	Lung	Endoscopic mucosal resection	Igawa et al. [10]
6	60	M	IDA	Double‐balloon enteroscopy	Small bowel	Cisplatin + vinorelbine ditartrate	25/11	N/A	Lung, brain	Segmental resection	Kunizaki et al. [11]
7	53	F	Abdominal pain, melena	Esophagogastroduodenoscopy	Duodenum, third portion	No treatment	7/0	N/A	Nil	N/A	Chung et al. [12]
8	60	M	Nil	Intraoperation incidental finding	Jejunum	No treatment	44/4	21	Lung, Spleen	Segmental resection	Iwaki et al. [13]
9	65	M	Intussusception	CT	Jejunum, 110 cm distal to the Treitz ligament	No treatment	0/2	N/A	Bone, Lymph node	Segmental resection	Kim et al. [6]
10	N/A	N/A	Elevating AFP and PIVKA II	^18^F‐FDG PET	Jejunum	No treatment	0/19	3	Lung	N/A	Sugiyama et al. [14]
11	64	M	Abdominal pain, stool OB(+)	Esophagogastroduodenoscopy	Duodenum	No treatment	4/0	2.2	N/A	N/A	Lin et al. [15]
12	69	F	—	Autopsy	Ileum	No treatment	N/A	—	Lung, Lymph node	—	Narita et al. [16]
13	36	M	Melena	Intraoperation	Jejunum	No treatment	0/0	0.5	Nil	Surgery	Chen et al. [17]
14	31	M	Occult GI bleeding	Barium study (intussusception)	Proximal jejunum	No treatment	19/0	N/A	Nil	Segmental resection	Yang et al. [18]
15	62	M	—	Autopsy	Small bowel	No treatment	N/A	—	Lung, Heart, Kidney, Bone, Skin	—	Tsujimoto et al. [19]

Abbreviations: AFP, alpha‐fetoprotein; CT, computed tomography; F, female; ^18^F‐FDG PET, ^18^F‐fluorodeoxyglucose positron emission tomography; GI, gastrointestinal; HCC, hepatocellular carcinoma; IDA, iron deficiency anemia; OB(+), occult blood positive; PIVKA II, prothrombin induced by vitamin K absence‐II; PVT, portal vein thrombosis.

^a^ From the initial diagnosis to first metastasis.

^b^ From the first metastasis to small bowel metastasis.

^c^ From small bowel metastasis to death or at the end of follow‐up (cases 2 and 8).

**TABLE 2:** Patients' characteristics among the 13 reported cases and the present case (*n* = 14).

		Number (%)
Age, years (mean ± SD)	58.9 ± 14.5	
Sex	Male	10 (71%)
	Female	3 (21%)
Symptom	Bleeding	7 (50%)
	Obstruction	3 (21%)
	Elevated tumor markers	6 (43%)
Initial staging	I	1 (7%)
	II	0 (0%)
	III	4 (29%)
	IV	5 (36%)
PVT	Positive	3 (21%)
	Negative	3 (21%)
AFP, ng/mL (mean ± SD)	At initial diagnosis (*n* = 6)	6831.2 ± 12871.1
	At small bowel metastasis (*n* = 5)	2140.0 ± 4361.8
PIVKA‐II, mAU/mL (mean ± SD)	At initial diagnosis (*n* = 1)	23 610
	At small bowel metastasis (*n* = 3)	15330.9 ± 25928.0
Morphology	Polypoid	5 (36%)
	Protruding mass	4 (29%)
	Ulcerative	3 (21%)
Treatment	Surgery	10 (71%)
	Endoscopy	1 (7%)

Abbreviations: AFP, alpha‐fetoprotein; CT, computed tomography; EGD, esophagogastroduodenoscopy; PET, positron emission tomography; PIVKA‐II, protein induced by vitamin K absence or antagonist‐II; PVT, portal vein thrombosis; SD, standard deviation.

The mean age at presentation was 58.9 years, and most (71%) patients were male. Most cases were diagnosed at a late stage (Stage III, *n* = 4; Stage IV, *n* = 5). Metastases were distributed across all three segments of the small intestine: duodenum (*n* = 3, 21%), jejunum (*n* = 5, 36%), and ileum (*n* = 5, 36%). With regard to symptoms, bleeding was the most common symptom, occurring in 50% (7/14) of patients: four cases of melena and positive fecal occult blood tests, two cases of iron deficiency anemia, and one case of a combination of these symptoms. Elevated levels of tumor markers, including AFP or PIVKA‐II, were reported in six patients before the diagnosis of small bowel metastasis and included three (21%) patients with no subjective symptoms, apart from elevated tumor markers, during regular follow‐up. Three (21%) patients developed acute‐onset small bowel obstruction and were diagnosed with intussusception. In one patient (Case 8), metastasis was incidentally found during surgery for other extrahepatic metastatic lesions.

The mechanism of hematogenous metastasis to the small intestine is thought to involve tumor invasion into the portal vein or tumor‐related portal vein thrombosis with subsequent tumor spread via hepatofugal flow. However, only five reports have documented the presence of portal vein thrombosis: three cases were positive and two cases were negative [6, 10, 12, 15, 16]. In our patient, the portal vein was patent at the initial diagnosis and at the latest follow‐up in March 2024. In our opinion, despite tumor macro‐invasion to the portal vein, patients with liver disease, including cirrhosis and portal hypertension, which are common in patients with HCC, tend to present with hepatofugal flow [20]. This factor may increase the possibility of micrometastasis of the primary tumor spreading to the gastrointestinal tract or parasplenic region (Case 8).

CT and ^18^F‐fluorodeoxyglucose positron emission tomography (^18^F‐FDG PET) are the most commonly used diagnostic tools in recent decades, whereas EGD is effective for identifying duodenal tumors. By contrast, cases reported before 2000 were less frequently diagnosed with these advanced imaging techniques. Capsule endoscopy and double‐balloon enteroscopy have also been used to detect and manage small bowel tumors (Cases 5 and 6). Metastatic tumors vary in morphology and include pedunculated or sessile lesions, submucosal masses, and ulcerative lesions. Synchronous multiple small bowel metastases were reported in three cases (Cases 9, 10, and 12). Tumor markers were documented in some patients and varied widely. Most recorded data were elevated at the initial diagnosis and at the time of small bowel metastasis.

Although small bowel metastasis from HCC is often a late‐stage phenomenon, with > 60% of reported cases presenting as Stage III or IV and only one case diagnosed at Stage I, our review revealed that 36% (5/14) of cases developed small bowel metastasis as one of the first extrahepatic sites. The interval between the initial HCC diagnosis and small bowel metastasis ranges widely from 0 to 76 months [8]. In our patient, the interval was 77 months, representing one of the longest durations among published cases [5, 7, 11, 13], which is likely attributable to advances in systemic treatment. However, surgical resection is still the most effective treatment for patients with good performance status and isolated extrahepatic metastases [21].

A previous report demonstrated that local endoscopic management of gastrointestinal bleeding without tumor resection had limited success, and all patients subsequently developed recurrent bleeding [15]. Most documented cases were treated surgically with segmental small bowel resection. One patient underwent endoscopic mucosal resection via double‐balloon enteroscopy (Case 5). Survival after small bowel metastasis was unfortunately reported in only six cases, and just one patient was followed for > 1 year [13], making treatment outcomes difficult to assess.

This review has several limitations, including the small number of reported cases and the broad time span of the literature (approximately 40 years). During this period, diagnostic modalities and treatment strategies evolved. Systemic therapy as the target therapy and immunotherapy were introduced and tested for these patients, potentially leading to different clinical decisions. Certain clinical details, such as the presence of portal vein thrombosis and tumor morphology, were not consistently documented in all cases. Nonetheless, to our knowledge, this paper is the most comprehensive review of hematogenous small bowel metastases from HCC. Clinicians should consider this rare presentation in the appropriate clinical scenarios.

## 4 Conclusion

Small intestine metastasis from HCC is rare but should be considered in patients presenting with unexplained gastrointestinal symptoms such as obstruction, occult bleeding, or elevated tumor marker levels. With advances in systemic therapy and improved survival, such atypical metastatic patterns may be encountered more frequently and warrant earlier surveillance. Surgical resection is the most effective treatment for small bowel metastases, with minimally invasive approaches and endoscopic resection options available for selected patients. Early detection and prompt treatment can be achieved with a better understanding of HCC metastasis to the small intestine.

## Author Contributions

Conceptualization: Meng‐Kai Hsu. Methodology: Meng‐Kai Hsu, Po‐Da Chen. Data Curation: Meng‐Kai Hsu. Formal analysis: Meng‐Kai Hsu, Po‐Da Chen. Investigation: Meng‐Kai Hsu, Po‐Da Chen. Resources: Yao‐Ming Wu. Writing – original draft: Meng‐Kai Hsu. Writing – review and editing: Meng‐Kai Hsu, Po‐Da Chen, Yao‐Ming Wu. Visualization: Meng‐Kai Hsu. Supervision: Po‐Da Chen, Yao‐Ming Wu. All authors have read and approved the final manuscript.

## Consent

Written informed consent was obtained from the patient for the publication of this case report and any accompanying images.

## Conflicts of Interest

The authors declare no conflicts of interest.

## Data Availability

The data generated and analyzed during the current study are not publicly available due to patient privacy restrictions but are available from the corresponding author on reasonable request.
